# Thigh muscle size and vascular function after blood flow-restricted elastic band training in older women

**DOI:** 10.18632/oncotarget.9564

**Published:** 2016-05-23

**Authors:** Tomohiro Yasuda, Kazuya Fukumura, Takanobu Tomaru, Toshiaki Nakajima

**Affiliations:** ^1^ Graduate School of Medicine, University of Tokyo, Tokyo, Japan; ^2^ School of Nursing, Seirei Christopher University, Shizuoka, Japan; ^3^ Faculty of Medicine, Toho University, Chiba, Japan; ^4^ Heart Center, Dokkyo Medical University Hospital, Tochigi, Japan

**Keywords:** sarcopenia, vascular occlusion, muscle hypertrophy, resistance exercise, arterial stiffness, Gerotarget

## Abstract

We examined the effect of elastic band training with blood flow restriction (BFR) on thigh muscle size and vascular function in older women. Older women were divided into three groups: low-intensity elastic band BFR training (BFR-Tr, *n* = 10), middleto high-intensity elastic band training (MH-Tr, *n* = 10), and no training (Ctrl, *n* = 10) groups. BFR-Tr and MH-Tr groups performed squat and knee extension exercises using elastic band, 2 days/week for 12 weeks. During BFR-Tr exercise session, subjects wore pressure cuffs around the most proximal region of both thighs. The following measurements were taken before (pre) and 3-5 days after (post) the final training session: MRI-measured muscle cross-sectional area (CSA) at mid-thigh, maximum voluntary isometric contraction (MVIC) of knee extension, central systolic blood pressure (c-SBP), central-augmentation index (c-AIx), cardio-ankle vascular index testing (CAVI), ankle-brachial pressure index (ABI). Quadriceps muscle CSA (6.9%) and knee extension MVIC (13.7%) were increased (*p* < 0.05) in the BFR-Tr group, but not in the MH-Tr and the Ctrl groups. Regarding c-SBP, c-AIx, CAVI and ABI, there were no changes between pre- and post- results among the three groups. Elastic band BFR training increases thigh muscle CSA as well as maximal muscle strength, but does not decrease vascular function in older women.

## INTRODUCTION

Skeletal muscle atrophy with aging (sarcopenia) inhibits mobility and increases the risk of falls, fractures, disability, and heart disease [1, 2]. High-intensity resistance training (HI-Tr, ≥70% 1-repetition maximum: 1RM) improves skeletal muscle morphology and function in young and older adults [3]. Since HI-Tr also improve insulin resistance and type-2 diabetes in the elderly [4, 5], the HI-Tr prevents and improves the sarcopenia in the elderly [6]. In general, traditional resistance exercise required for weight machines/free weights, but this method is not be practical and may even be dangerous. Thus, the effectiveness of alternative exercise methods should be investigated.

Elastic bands/tubing have been used practically (portable, less expensive, easy to used) in rehabilitative medicine and in health enhancement for resistance training [7, 8]. Additionally, home-based resistance-training program for older adults using elastic bands improves muscle strength [9]. However, since elastic resistance training is commonly performed using a low-to-moderate resistance level, this training typically has little or no effect on muscle hypertrophy [9, 10].

In the past 15 years, several studies revealed that muscle hypertrophy can be produced with low-intensity resistance training (~30% 1RM) with blood flow restriction (BFR-Tr) regardless of age [11, 12]. This suggests that BFR-Tr using elastic bands for resistance may be an effective home-based resistance training program for promoting both muscle hypertrophy and strength in older adults. Unfortunately, low-intensity elastic band BFR training-induced muscle hypertrophy gain are not fully understood.

In general, prevention and treatment for decreased arterial compliance or stiffness is important [13,14]. Previous studies are split on whether HI-Tr decrease [15,16] arterial compliance in young and older adults or not [17,18]. This means that the HI-Tr has a potentially decrease regarding arterial function in older adults. On the other hand, BFR-Tr could improve or maintain arterial function in young and older adults [19-21]. Therefore, we hypothesized that BFR-Tr produces muscle hypertrophy with a low risk of increased arterial stiffness in older adults.

Sarcopenia is muscle specific and that greater quadriceps muscle loss was found in older adults [22,23]. Thus, the purpose of this study was to examine the effect of low-intensity elastic band training with BFR on thigh muscle size and arterial stiffness in older adults.

## RESULTS

Before training, there were no significant differences among three groups for age (*p* = 0.383) and anthropometric variables (standing height, *p* = 0.125; body mass, *p* = 0.783; BMI, *p* = 0.311; mid-thigh girth, *p* = 0.195; lower leg girth, *p* = 0.257), MVIC (knee extension, *p* = 0.229; knee flexion, *p* = 0.363), muscle CSA (quadriceps, *p* = 0.122; adductors, *p* = 0.218; hamstrings, *p* = 0.148; gluteus maximus, *p* = 0.540) (Table 1), hemodynamic parameter and vascular function (*p* = 0.458-0.941), coagulation system and creatine kinase (*p* = 0.251-0.321) except for leg press 1RM strength (*p* = 0.044) and D-dimer (*p* = 0.029) (Table 2). A significant group-by-time interaction was not observed for body weight (*p* = 0.655) and BMI (*p* = 0.928) in three groups following the training period.

**Table 1:** Changes in anthropometric variables after 12 weeks of training period

	BFR-Tr			MH-Tr			Ctrl		
	Pre	Post	%	Pre	Post	%	Pre	Post	%
Anthropometric variables									
Age, yrs	70 (6)			72 (7)			68 (6)		
Standing height, m	1.53 (0.06)			1.52 (0.06)			1.57 (0.07)		
Body mass, kg	48.3 (5.8)	48.4 (5.9)	0.0	48.7 (8.1)	48.3 (7.2)	−0.7	55.0 (7.1)	54.8 (6.9)	−0.4
BMI, kg/m^2^	20.8 (2.5)	20.7 (1.9)	0.2	20.9 (2.1)	20.7 (1.8)	−0.7	22.3 (2.8)	22.2 (2.7)	−0.4
Mid-thigh girth, cm	43.9 (3.8)	44.2 (3.7)	0.7	45.1 (4.0)	44.9 (4.1)	−0.4	46.9 (2.8)	46.4 (3.1)	−1.1
Lower leg girth, cm	32.8 (2.3)	32.9 (2.1)	0.3	32.6 (3.3)	32.8 (3.2)	0.6	34.4 (1.8)	34.2 (1.9)	−0.4

Notes: Data are given as mean (±SD). BFR-Tr = low-intensity elastic band BFR training; MH-Tr = middle-to-high-intensity elastic band training; Ctrl = non-BFR resistance training; BMI = body mass index; Mid-thigh girth = at 50% thigh length; Lower leg girth = at 30% lower leg length.

**Table 2:** Changes in hemodynamic parameter and vascular function, coagulation system and muscle damage after 12 weeks of training period

	BFR-Tr		MH-Tr		Ctrl	
	Pre	Post	Pre	Post	Pre	Post
Hemodynamic parameter and vascular function						
Resting heart rate, bpm	67 (17)	65 (11)	65 (7)	67 (7)	64 (4)	62 (6)
c-SBP, mmHg	138 (20)	131 (15)	138 (29)	122 (9)	134 (12)	128 (20)
c-AIx, %	84 (11)	89 (12)	84 (19)	86 (15)	86 (11)	88 (13)
b-SBP, mmHg	132 (20)	125 (26)	130 (18)	125 (18)	125 (10)	120 (12)
b-DBP, mmHg	78 (11)	75 (26)	81 (8)	78 (9)	79 (8)	79 (8)
a-SBP, mmHg	152 (24)	148 (40)	145 (19)	141 (20)	142 (17)	141 (16)
a-DBP, mmHg	74 (10)	73 (9)	75 (8)	75 (8)	73 (7)	75 (7)
CAVI, m/sec	8.4 (0.9)	8.5 (0.8)	8.4 (0.9)	8.5 (0.8)	8.3 (0.8)	8.4 (0.7)
ABI, unit	1.14 (0.06)	1.15 (0.04)	1.11 (0.05)	1.11 (0.08)	1.11 (0.06)	1.12 (0.05)
Coagulation system						
FDP, μg/dl	3.2 (1.0)	3.6 (2.1)	4.1 (1.9)	5.0 (3.6)	3.2 (0.9)	2.7 (0.9)
D-dimer, μg/dl	0.4 (0.2)	0.5 (0.3)	0.2 (0.2)	0.3 (0.1)	0.2 (0.1)	0.4 (0.2)
Muscle damage						
CK, IU/l	109 (70)	91 (31)	93 (31)	99 (41)	130 (55)	95 (24)

Notes: Data are given as mean (±SD). BFR-Tr = low-intensity elastic band BFR training; MH-Tr = middle-to-high-intensity elastic band training; Ctrl = non-BFR resistance training; c-SBP = central systolic blood pressure; c-AIx = central augumentation index; b-SBP = brachial systolic blood pressure; b-DBP = brachial diastolic blood pressure; CAVI, cardio-ankle vascular index; ABI, ankle-brachial pressure index; FDP, fibrin/fibrinogen degradation products; CK, creatine kinase.

### Acute effect of BFR-Tr and MH-Tr

**Figure 1:**
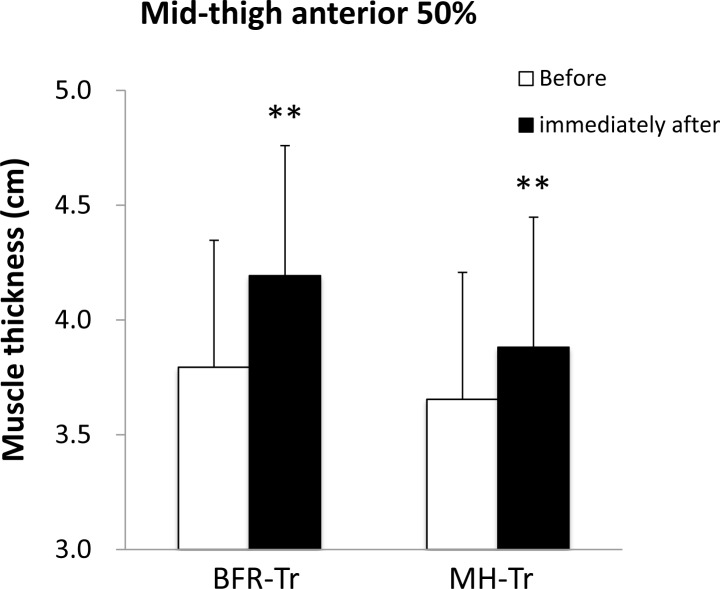
Muscle thickness of mid-thigh at anterior 50% before and immediately after exercise session Data are given as mean (± SD). **Different from before, *P* < 0.01.

A significant group by time interaction was observed for anterior mid-thigh (*p* = 0.032) but not for posterior mid-thigh MTH (*p* = 0.731). Immediately after the exercise session, mean MTH was increased in both BFR-Tr and MH-Tr groups (*p* = 0.001 and *p* = 0.002, respectively), and the percent change in anterior mid-thigh MTH tended to be greater (*p* = 0.062) in the BFR-Tr group (pre: 3.8 (0.6) cm *vs*. post: 4.2 (0.6) cm: 10.6%) compared with MH-Tr group (pre: 3.7 (0.5) cm *vs*. post: 3.9 (0.6) cm: 6.3%) (Figure 1).

There were no differences between BFR-Tr and MH-Tr groups in the elastic band elongation (*p* = 0.734 for squatting and *p* = 0.738 for knee extension, respectively) (Table 3), ratings of perceived exertion (*p* = 0.075 for squatting and *p* = 0.341 for knee extension, respectively), and range of motion (*p* = 0.073 (knee joint) and *p* = 0.311 (hip joint) for squatting and *p* = 0.387 for knee extension, respectively). Heart rate was higher in the BFR-Tr group than in the MH-Tr group for squatting (*p* = 0.515), but not for knee extension (*p* = 0.047) (Table 4).

**Table 3:** Elastic band elongation during two exercises

	BFR-Tr		MH-Tr	
	Flexed position	Extended position	Flexed position	Extended position
Elastic band elongation, cm				
Squat				
12^th^ training session	0.9 (4.1)	38.9 (3.8)	2.7 (5.3)	40.3 (3.4)
24^th^ training session	4.5 (3.7)	46.4 (4.4)	2.7 (4.8)	45.7 (3.6)
Knee extension				
12^th^ training session	8.4 (4.5)	44.9 (4.7)	6.3 (4.3)	42.5 (7.0)
24^th^ training session	14.2 (3.6)*	52.2 (5.7)	9.3 (4.1)	46.1 (7.6)

Notes: Data are given as mean (±SD). BFR-Tr = low-intensity elastic band BFR training; MH-Tr = middle-to-high-intensity elastic band training.

* p<0.05, BFR-Tr versus MH-Tr.

**Table 4:** Ratings perceived exertion, heart rate and range of motion during two exercises

	BFR-Tr		MH-Tr	
	Mean (±SD)	Range	Mean (±SD)	Range
Ratings of perceived exertion, OMNI-Res Scale				
Squat				
12^th^ training session	7.7 (1.4)*	6-10	5.8 (1.5)	5-7
24^th^ training session	7.2 (1.4)	6-10	6.4 (1.0)	6-8
Knee extension				
12^th^ training session	8.2 (1.4)*	6-10	7.0 (0.9)	6-8
24^th^ training session	8.0 (1.4)	6-10	7.5 (1.3)	6-9
Heart rate, bpm				
Squat				
12^th^ training session	129 (21)	101-167	119 (19)	82-150
24^th^ training session	129 (22)	96-164	129 (16)	107-155
Knee extension				
12^th^ training session	130 (25)	93-167	108 (19)	87-141
24^th^ training session	123 (20)	94-166	111 (12)	92-128
Range of motion, degree				
Squat				
Hip joint				
12^th^ training session	69 (8)	60-81	67 (16)	41-92
24^th^ training session	66 (11)	52-86	59 (11)	45-81
Knee joint				
12^th^ training session	81 (15)	61-101	96 (22)	62-129
24^th^ training session	93 (16)	73-126	106 (17)	75-134
Knee extension				
Knee joint				
12^th^ training session	80 (6)	75-90	84 (6)	73-94
24^th^ training session	85 (5)	76-95	85 (12)	70-104

Notes: Data are given as mean (±SD). BFR-Tr = low-intensity elastic band BFR training; MH-Tr = middle-to-high-intensity elastic band training.

* p<0.05, BFR-Tr versus MH-Tr.

### Chronic effect of BFR-Tr and MH-Tr

**Figure 2:**
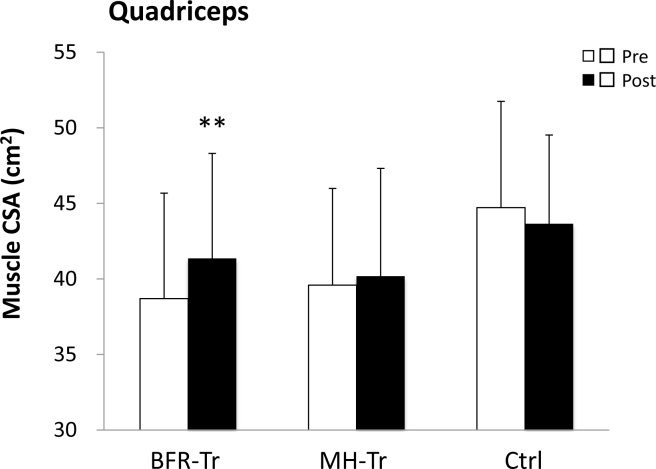
Muscle cross-sectional area (CSA) of quadriceps pre- and post-training period Data are given as mean (± SD). **Different from pretraining, *P* < 0.01.

**Figure 3:**
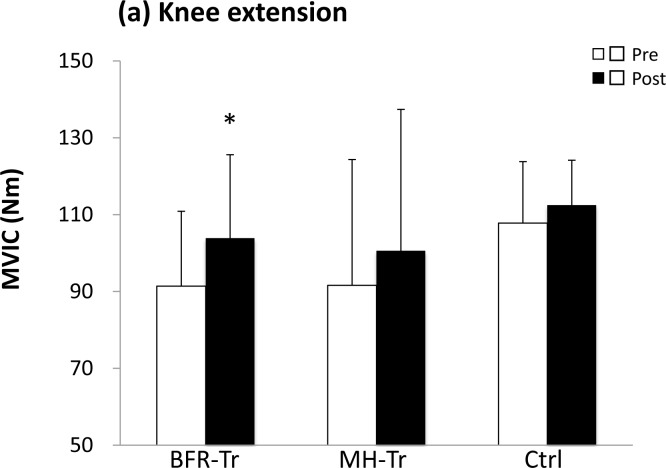

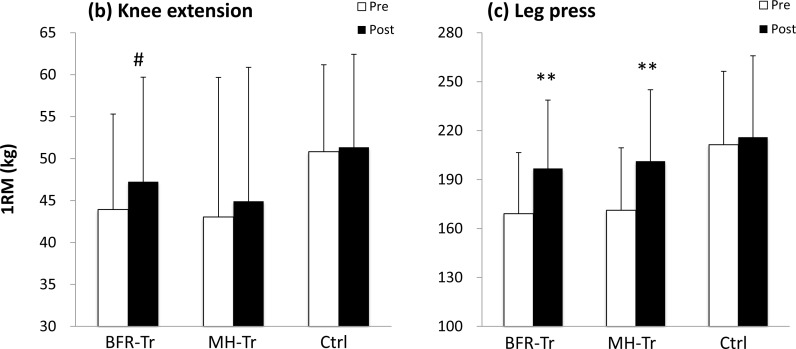
Maximum voluntary isometric contraction (MVIC) of knee extension **a.** and 1RM of knee extension **b.** and leg press **c.** pre- and post- training period. **Different from pretraining, *P* < 0.01. *Different from pretraining *P* < 0.05, ^#^Different from pretraining, *P* = 0.08.

A significant group by time interaction was observed for muscle CSA (*p* < 0.001 for quadriceps), MVIC (*p* = 0.028 for knee extension) and leg press 1RM (*p* < 0.001), but not for knee extension 1RM (*p* = 0.076). Quadriceps muscle CSA (6.9%) was increased (*p* < 0.001) in the BFR-Tr group, but not in the MH-Tr (1.5%, *p* = 0.871) and the Ctrl (−2.2%, *p* = 0.395) groups (Figure 2). Knee extension MVIC (13.7%) was increased (*p* = 0.028) in the BFR-RT group, but not in the MH-Tr (*p* = 0.196) or Ctrl (*p* = 0.810) groups (Figure 3a). Knee extension 1RM (7.6%) tended to be increased (*p* = 0.076) in the BFR-RT group, but not in the MH-Tr (*p* = 0.605) or Ctrl (*p* = 0.998) groups (Figure 3b). Leg press 1RM (16.4% and 17.6%) was increased (both *p* < 0.001) in the BFR-RT and the MH-Tr groups, respectively, but not in the Ctrl (*p* = 0.912) group (Figure 3c). The magnitude of effect size between pre- and post-training was larger in the BFR-Tr group than in the MH-Tr group for quadriceps muscle CSA (small *vs*. trivial), knee extension MVIC (moderate *vs*. small), and knee extension 1RM (small *vs*. trivial), but not for leg press 1RM (moderate *vs*. moderate) (Table 5).

**Table 5:** Effect size in muscle size and strength after 12 week of training period

	BFR-Tr	MH-Tr	Ctrl
	Pre to post	Pre to post	Pre to post
Muscle CSA			
Quadriceps	0.38^a^	0.09	−0.15
Adductors	−0.05	0.10	−0.23
Hamstrings	0.03	−0.02	−0.25
Gluteus maximus	0.14	0.11	−0.01
MVIC			
Knee extension	0.64^b^	0.27^a^	0.29^a^
Knee flexion	0.00	0.03	−0.24
1RM			
Knee extension	0.29^a^	0.11	0.05
Leg press	0.74^b^	0.79^b^	0.10

Notes: Data are given as mean (±SD). BFR-Tr = low-intensity elastic band BFR training; MH-Tr = middle-to-high-intensity elastic band training; Ctrl = non-BFR resistance training; CSA = cross-sectional area; MVIC = maximal voluntary isometric contraction; 1RM = one-repetition maximum.

a = small effect size

b = moderate effect size.

No significant group-by-time interaction was observed for muscle CSA (*p* = 0.104 for adductors, *p* = 0.119 for hamstrings, and *p* = 0.623 for gluteus maximus), MVIC (*p* = 0.419 for knee flexors), hemodynamic parameter and vascular function (*p* = 0.474-0.929), coagulation system (*p* = 0.599-0.753), and creatine kinase (*p* = 0.099) (Table 2).

## DISCUSSION

BFR-Tr using weight machines/free weight leads to increased thigh muscle size and the maintenance of arterial stiffness [20,21]. In this study, low-load, elastic band resistance training (squatting and knee extension) with BFR can leads to increase in quadriceps muscle CSA as well as maximal strength in older women. In addition, the hemodynamic parameter, vascular function, coagulation system and creatine kinase are no changes.

In this study, MH-Tr group did not increase muscle hypertrophy for lower limb. Previous study [24] reported that elastic band MH-Tr is not easy to induce the muscle hypertrophy even for upper limbs. Since resistance level by elastic band is required greater in lower limb compared with upper limb, it is increasingly difficult to control in direction for resistance, ROM for active muscles, and fixation for handgrip. On the other hand, the BFR-Tr group increased in quadriceps muscle CSA. Additionally, the observed gain in quadriceps muscle CSA (0.29% per session) provided comparable results as BFR training using free weights (0.33% per session) [21]. Thus, low-intensity elastic band training with BFR can provide a sufficient hypertrophic stimulus for thigh muscles in older adults.

There are some trigger mechanisms underlying the BFR-Tr-induced muscle hypertrophy. In HI-Tr, myogenic stem cells most likely activate muscle protein synthesis [25]. BFR-Tr leads to proliferation of myogenic stem cells, an increase of myonuclei in skeletal muscle, and marked myofiber hypertrophy [26]. Therefore, myogenic stem cell-derived myonuclei may provide an improved capacity for myofibrillar gene transcription, which enhanced activity of muscle protein synthesis for BFR-Tr by using elastic bands as well as weight machines and/or free weights.

BFR-Tr-induced muscle cell swelling may activates to the anabolic benefits of BFR [27, 28, 29]. With respect to effect size for acute MTH, an index of muscle cell swelling, of the anterior thigh, the BFR-Tr group showed a greater MTH response compared with the MH-Tr group (0.72 *vs*. 0.41). It is known that acute cell swelling simulates anabolic processes through an increase in protein synthesis and a decrease in proteolysis [30, 31]. Thus, the BFR exercise-induced enhancement of muscle protein metabolism may increase in quadriceps muscle size.

In the BFR-Tr group, increased muscle strength (%change per session and effect size) in this study was lower than that in the previous study [21] for knee extension (0.32% *vs*. 1.09% and 0.29 *vs*. 0.70) and leg press 1RM (0.68% *vs*. 1.39% and 0.74 *vs*. 0.98). In general, elastic band training did not large stimulation in the 1RM strength because resistance type is dramatic difference between elastic bands and weight machines. Thus, it appears that the difference in improvement in muscle strength between elastic bands and weight machines is attributed to unequal neural adaptations.

An elastic band resistance training is well tolerated, as indicated by non-exacerbation of chronic disease conditions and lack of training-induced injury [8,9]. In this study, the ratings of perceived exertion were higher in the BFR-Tr group compared with the MH-Tr group, but the hemodynamic parameter, vascular function, coagulation system and creatine kinase were not changed for the both groups. In particular, central blood pressure and cardiac afterload were not increased by BFR-Tr group as well as MH-Tr group. In general, endothelial function, as assessed by brachial flow-mediated dilatation (FMD), has an inverse relationship with central arterial stiffness [32]. FMD was not assessed in this study, but previous study reported that FMD was not decreased by 12 weeks of BFR-Tr [21]. This may explain why there was not an increase in central blood pressure and cardiac afterload following BFR-Tr. Together, BFR-Tr using elastic bands is a relatively safe training method. However, it should be noted that the possibility of side effects cannot be denied when subjects perform such training until near exhaustion or particularly to complete exhaustion [33,34].

The present study has some limitations. First, it should be noted that our sample size was small. In this study, the effect size in muscle CSA (quadriceps: 0.38 for BFR-Tr), MVIC (knee extension: 0.64 for BFR-Tr and 0.27 for MH-Tr) and 1RM (knee extension: 0.29 for BFR-Tr, leg press: 0.74 for BFR-Tr and 0.79 for MH-Tr) following the training period was not high (low-to-moderate level of effect size). Totally, the effects of elastic bands BFR-Tr on muscle size and strength was small compared with weight machines BFR-Tr, although the training protocol was basically similar between the two studies (this study *vs*. previous study [21]). Second, it was very difficult to evaluate the exercise intensity using elastic bands. As in the case of the previous study [24], we employed a similar method by adjusting the distance of the band stretch based on OMNI-Res scale [35], but the precise exercise intensity was merely estimated. Third, the type of band used in our study presented close to the same resistance level (gold, black, etc.) for each group, consequently the arbitrary pressure was used. Forth, since post-training measurements were performed 3-7 days, the difference of measurement date might suppress the results. Lastly, because our participants were only old women, it is uncertain whether the results pertain to old men. Additional research into these issues is needed.

In conclusion, low-intensity squatting and knee extension training with BFR using elastic bands for resistance elicited marked gains in quadriceps muscle CSA and strength, and did not decrease vascular function in older women. Thus, our results demonstrate that low-intensity, elastic band BFR training would be beneficial in the development of safe and effective methods of sarcopenia care and prevention in older adults.

## MATERIALS AND METHODS

### Participants

Thirty women (aged 61-86 years) volunteered to participate in the study and were selected according to the exclusion criteria (blood pressure >160/100 mmHg, body mass index >30 kg/m^2^, history of anemia, cerebrovascular disease, myocardial infarction and arthroscopic joint surgery) used to define “medically stable” older participants for exercise studies proposed by Greig et al. [36]. In addition, volunteers who suffered from a chronic disease such as severe hypertension (>170/110 mmHg), orthopedic disorders, deep venous thrombosis, peripheral vascular disease, or cognitive dysfunction were excluded from the study. The participants in this study were physically active, with nine (BFR-Tr, *n* = 3; MH-Tr, *n* = 4; Ctrl, *n* = 2) of 30 participated in regular aerobic-type exercise (walking, jogging or cycling; 2-3 times per week for approximately 30 min in duration). None of the participants had participated in resistance-type training for a minimum of 6 months prior to the study. All participants were non-smokers, were free of overt chronic disease as assessed by medical history, physical examination, and complete chemistry and hematologic evaluation. No participant was on hormonal replacement therapy. They were randomly divided into a low-intensity elastic band BFR training (BFR-Tr, n = 10, mean ± SD: 70 ± 6 years) or a middle-to-high-intensity elastic band training (MH-Tr, n = 10, 72 ± 7 years) or no training (Ctrl, n = 10, 68 ± 6 years) groups. Eight (BFR-Tr, n = 3; MH-Tr, n = 3; Ctrl, n = 2) of all participants were classified as “hypertension (>140/90 mmHg)” [37], but nobody took medicine for high blood pressure. All participants were informed of the risks associated with involvement in the study and signed an informed consent document before participation. The principles of the World Medical Association Declaration of Helsinki and the American College of Sports Medicine Guidelines for Use of Human Subjects were adopted in this study. The study was approved by the Ethics Committee of the University of Tokyo.

### Training protocol

**Figure 4:**
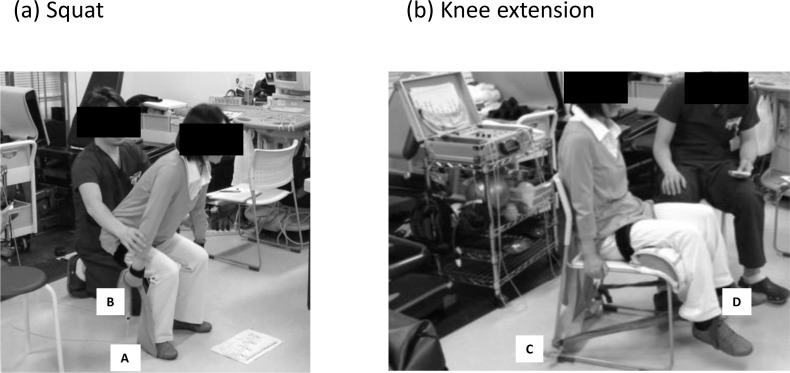
Illustrations show the body position used for two exercises Squat **a.** and knee extension **b.** exercises were performed with an elastic band. A = outside foot; B = grip; C = tie on posterior chair leg; D = ankle.

Two training groups performed bilateral squat and knee extension exercise training 2 days/week for 12 weeks (Figure 4). The MH-Tr group exercised at two exercise intensities ranging from 5.6 to 8.4 on the OMNI perceived exertion scale for resistance exercise (OMNI-RES) for active muscle scale (0-extremely easy to 10-extremely hard) which has been noted to correspond to exercise intensity levels ranging from approximately 70% to 90% of 1RM for women [35]. If the intensity range was below 5 or above 9, the intensity of the two exercises was modified to either increase or decrease the resting length of the elastic band. As a result of the pilot study, the MH-Tr groups used two gold (Max) bands for squatting, and one gold band for knee extension (Hygenic Corporation, Akron, Ohio, USA).

The BFR-Tr group used one gold band for squatting, and one black (Special Heavy) band for knee extension. The gold band was approximately twice the resistance level as the black band [38], thus the two exercises for the BFR-Tr group were one-half (low-intensity level) the intensity as that for the MH-Tr group. These training exercises were performed under the close supervision of those with technical knowledge in BFR training. The BFR-Tr group exercised at two exercise intensities ranging from 5 to 9 on the OMNI Resistance for active muscle scale. If the intensity range was below 5 or above 9, the intensity of the two exercises was modified to either increase or decrease the resting length of the elastic band and/or restriction pressure intensity. In both groups, the repetition duration was 2.6 seconds (1.3-second concentric and 1.3-second eccentric exercise cycle) and 2.0 seconds (1.0-second concentric and 1.0-second eccentric exercise cycle) for squat and knee extension, respectively.

One week before the start of the training study, both groups performed practice sessions for the maximum voluntary isometric contraction (MVIC) and knee extension and leg press 1RM test. In addition, the BFR-Tr participants became familiar with the BFR stimulus. Three or four days before training, the MVIC and 1RM were determined. In the BFR-Tr group, training was set at 75 repetitions (30, 15, 15, and 15 repetitions, with a 30-second resting period between sets) for both exercises (90-second rest between exercises). This protocol is consistent with submaximal BFR studies [12,20,21,39]. Once the pneumatic cuffs were inflated, they remained inflated for the two exercises, including during the resting periods between sets and exercises. On the other hand, in the MH-Tr group, training was set at 37 or 38 repetitions (13, 13 (at 1^st^ - 12^th^ training session) or 12 (at 13^th^ - 24^th^ training session), and 12 repetitions with 30-second rests between sets) for both exercises (90-second rests between exercises). Exercise repetition for BFR-Tr group was twice that for MH-Tr group, although the exercise intensities for MH-Tr group were twice that for BFR-Tr group. Therefore, the same training volume (exercise intensity X repetitions) was set between the two training groups.

**Figure 5:**
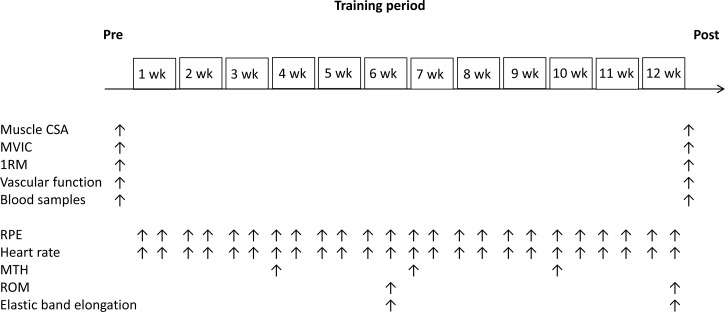
Experimental timeline CSA = cross-sectional area; MVIC = Maximum voluntary isometric contraction; 1RM = one repetition maximum; RPE = ratings of perceived exertion; MTH = muscle thickness; ROM = range of motion.

During squat exercises, knee joint range of motion (ROM) and hip joint ROM were approximately 100-0 degrees and 100-0 degrees, respectively, while during knee extension exercises, knee joint ROM was approximately 95-15° and hip joint ROM was maintained at 90° (with 180° being full extension). Subjects were instructed not to let the band snap them back to the start position but rather to consciously control the return movement such that it would take twice as long as the stretching movement. In addition, elastic band elongation was measured by tape measure for squat (distance from outside foot [a] to grip [b]) and knee extension (distance from tie on posterior chair leg [c] to ankle [d]) exercises (Figure 4). The measurements of ROM and elastic band elongation were completed at the 6^th^ week and 12^th^ week of the training period (Figure 5).

### Blood flow restriction

During the training sessions, BFR-Tr participants wore a specially designed pneumatic cuff (50 mm width, KAATSU Master, KAATSU Japan Co., Ltd., Tokyo, Japan) around the most proximal portion of both thighs. On the first day of training, the cuffs were set at 50 mmHg and air pressure was gradually inflated to 120 mmHg (Day 1). The air pressure was increased by 10-20 mmHg at each subsequent training session until a pressure of approximately 200 mmHg was reached if the subject could perform at a high level of pressure intensity. The restriction pressure was selected in accordance with a previous study [40]. The mean pressure intensity throughout the period of training was 161 ± 12 mmHg (160-200 mmHg at 24^th^ training session). Immediately after the two exercises, the pressure cuff was quickly removed. The amount of time under blood flow restriction was approximately 10-11 min.

### Measurements schedule

Subject testing took place before the start of the study (pre) and 3-7 days after (post) the 12-week training period. The order of measurements were MRI, venous blood samples, vascular function tests [central systolic blood pressure (c-SBP), central-augmentation index (c-AIx), cardio-ankle vascular index testing (CAVI), ankle brachial pressure index (ABI)], maximum voluntary isometric contraction (MVIC) and 10RM strength. Figure 5 shows the testing schedule for each of the measurements taken during the 12 week experimental period. All data were obtained from the right side of the body. Considering the schedule of examiners, all subjects and variable technologists, the MRI (9:00 and 15:00 hours), venous blood samples and arterial function tests (after 6-7 hours of fasting, 9:00 and 15:00 hours), and MVIC (9:00 and 15:00 hours) measurements were obtained on two days. The subjects were instructed to not drink alcohol or caffeine during the 24-hour period and from performing any strenuous exercise during the 48-hour period prior to pre- and post-training measurements.

### MRI-measured muscle CSA

Muscle CSA was obtained using a MRI scanner (0.2-T Open MRI, Hitachi, Tokyo, Japan). A T-1 weighted, spin-echo, axial plane sequence was performed with a 500-msec repetition time and a 23-msec echo time. Subjects rested quietly in the magnet bore in a supine position with their legs extended. The top edge of the great trochanter was used as the origin point, and continuous transverse images with 10-mm slice thickness (0-mm interslice gap) were obtained from the top edge of the great trochanter to the lateral condyle of femur at pre- and post-training measurements. All MRI data were transferred to a personal computer for analysis using specially designed image analysis software (sliceOmatic, Tomovision Inc., Magog, QC, Canada). Skeletal muscle tissue cross-sectional area (CSA) data for the quadriceps, adductors and hamstrings at 50% of thigh length and for the gluteus maximus at the top edge of the great trochanter were digitized. The coefficient of variation of this measurement was less than 1.0% [21].

### Maximum voluntary isometric contraction

MVIC of the knee extensors and flexors was determined by using a dynamometer (Biodex System 3, Sakai Medical Instrument, Tokyo, Japan). Participants were carefully instructed so that they became familiarized with the testing procedures of voluntary force production of the thigh muscles during several submaximal and maximal performances about 1 week before testing. The participants were seated on a chair with their hip joint angle positioned at 85° (0° at full extension). The center of rotation of the knee joint was visually aligned with the axis of the lever arm of the dynamometer and the ankle of the right leg was firmly attached to the lever arm of the dynamometer with a strap. Several warm-up contractions were performed before testing. Participants were then instructed to perform maximal isometric knee extension and flexion at a knee joint angle of 80° and 40° (0° at full extension), respectively. If MVIC torque for the first two MVICs (60-sec rest interval) varied by more than 5%, up to two additional MVICs were performed with a 60-sec rest between trials. Participants were instructed to perform an MVIC as quickly as possible during a period of about 2 seconds. The highest MVIC value was used for data analysis. The test-retest reliability (ICC, SEM and minimal difference) was previously determined using the data of 9 older women measured twice within 7 days (at least one day apart) for MVIC (0.917, 5.03 Nm, 13.9 Nm).

### Estimation of 1RM Strength

One RM was estimated by the 10RM method (1-RM = 100 · rep mass / (48.8 + 53.8 ·exp [−0.075 · reps]) [41] using a weight stack machine. Bilateral knee extension and leg press maximum dynamic strength (1RM) were assessed using an isotonic knee extension equipment (VR1, Cybex International, Inc.) and a leg press machine (Seated Leg Press, Life Fitness). After warming up, the testing load was set (approximately 80% of predicted 1RM). Each participant reached muscular failure for the load, and partial repetitions (where participants failed to lift through the entire ROM) did not count as RMs. If a participant had to perform a given repetition number for a given condition again, as a result of ease in obtaining the desired repetitions or failure to attain the repetition number, a 5-minute rest period was given and the condition was attempted again at an altered load. No participant had to perform a given repetition number test condition more than 3 times. Each participant performed the knee extension exercise, rested for 5 minutes, and then performed the leg press exercise. During estimated 1RM testing as well as training sessions, the parallel leg stance width was set at 100% of the shoulder-width for leg press exercises. The test-retest reliability (ICC, SEM and minimal difference) was previously determined using the data of 9 older women measured twice within 7 days (at least one day apart): 0.990, 1.61 kg, 4.47 kg for knee extension 1RM and 0.990, 3.44, 9.53 for leg press.

### Vascular function tests

c-SBP and c-AIx, measurements were conducted in the seated position. The participants were instructed to fast 6-7 hours before testing and refrain from alcohol or caffeine intake for at least 12 hours prior to testing. The c-SBP was examined in the seated position in a quiet temperature-controlled room (25-26°C). Radial artery pressure waveforms and brachial BP were recorded simultaneously using a fully automated device (HEM-9000AI, Omron Healthcare Co., Ltd., Kyoto, Japan) to calculate late systolic pressure in the radial artery and to estimate c-SBP and c-AIx. There were significant positive correlations between aorta-SBP and radial-SBP (*r* = 0.95, *p* < 0.001) or aorta-AIx and radial-AIx (*r* = 0.91, *p* < 0.001) [42]. The brachial BP was measured with an oscillometric manometer and the radial pulse waveforms were recorded noninvasively using an applanation tonometer, which consisted of a sensor unit with an array of 40 fine-pitch microtransducer elements and a monitor unit [43]. Then, CAVI and ABI were measured noninvasively using a VS-1500 system (Fukuda Denshi Co., Ltd., Tokyo, Japan). The CAVI and ABI measurements were conducted in the supine position. Participants were asked to rest in a quiet temperature-controlled room (25-26°C) for 20-30 min. Electrocardiogram and heart sound were monitored. CAVI was automatically calculated using the formula; a {2ρ/ΔP x In(Ps/Pd)PWV^2^} + b (a and b, constants; ρ, blood density; P, difference in systolic and diastolic pressure; Ps, systolic pressure wave velocity; Pd, diastolic pressure; PWV, heart-ankle pulse wave velocity). The ankle-brachial pressure index (ABI) was calculated as the ratio of the systolic blood pressure in the ankle to the systolic blood pressure in the right arm [44]. To measure the stiffness the aorta, CAVI is essentially independent of blood pressure unlike PWV [44]. Therefore, arterial function was determined using CAVI. The test-retest reliability (ICC, SEM and minimal difference) was previously determined using the data of 9 older women measured twice within 7 days (at least one day apart): 0.974, 4.12 mmHg, 11.4 mmHg for c-SBP, 0.823, 2.52 %, 6.98 % for c-AIx, 0.957, 0.15 m/sec, 0.42 m/sec for CAVI and 0.439, 0.04 unit, 0.10 unit for ABI.

### Blood sampling and biochemical analyses

Venous blood samples were obtained from the antecubital vein and measured for fibrin/fibrinogen degradation products (FDP), D-dimer and creatine kinase (CK). The plasma concentrations of these samples were measured at a commercial laboratory (SRL Inc., Tokyo, Japan) by following latex immunoassay (LIA) for FDP and D-dimer and spectrophotometry for NADPH formed by a hexokinase and D-glucose-6-phosphate-dehydrogenase-coupled enzymatic system for CK.

### Measurements of acute responses to training session

### Ratings of perceived exertion

During all training sessions, OMNI-RES based on a numerical scale of 0 to 10 were collected to assess subjective feelings of physical effort (i.e. exertion). OMNI-RES data were recorded immediately after the last set of each exercise [35].

### Ultrasound-measured muscle thickness

Since muscle thickness (MTH) measurement using a B-mode ultrasound (Acuson Sequoia 512, Siemens, Tokyo, Japan) has the advantage of evaluating acute change in muscle cell swelling following exercises [29], the following measurements were made: MTH of the anterior and posterior mid-thigh, midway between the lateral condyle of the femur and greater trochanter; and posterior lower leg, at 30% proximal between the lateral malleolus of the fibula and the lateral condyle of the tibia. Briefly, the measurements were carried out while the subjects stood with their elbows extended and relaxed. A 10.0 MHz scanning head (5.5 cm length probe) was placed on the skin perpendicular to the tissue interface. The scanning head was coated with a water-soluble transmission gel to provide acoustic contact without depressing the dermal surface. The subcutaneous adipose tissue-muscle interface and the muscle-bone interface were identified from the ultrasonic image. The perpendicular distance from the adipose tissue-muscle interface to the muscle-bone interface was considered to represent MTH. Ink markers on the anterior and posterior thigh and posterior lower leg were used to ensure similar positioning over repeated MTH measurements. The MTH was recorded before and immediately after the exercise bout. This measurement was completed at the 7^th^ week and 10^th^ week of the training period, and the average of two measurements for “before” or “immediately after” was represented as a single data point for statistical analysis, respectively. The test-retest reliability (ICC, SEM and minimal difference) was previously determined using the data of 9 older women measured twice within few days for MTH (0.994, 0.28 cm, 0.79 cm).

### Heart rate

During all training sessions, heart rate was recorded at baseline (pre) and immediately after the last set of each exercise (post) (Model 9560, Onyx II, Nonin Medical Inc., Plymouth, MN, USA).

### Statistical analyses

The results are expressed as mean ± standard deviation (SD). The data were tested for normality using the Shapiro-Wilk test. Because all variables were normally distributed, parametric statistical analyses were performed. Two-way ANOVA with repeated measures [condition (BFR-Tr, MH-Tr, Ctrl) by time (pre, post)] was used to evaluate the training effects for all dependent variables. When significant main effects and/or interaction were observed, post hoc testing was performed using Tukey post hoc test. Statistical significance was set at *p* < 0.05. The sample size was estimated from a priori power analysis [45] to detect differences (power of 0.80 an α of 0.05, two-tailed, and an effect size of 1.6-2.1) in knee extension and leg press 1RM (*n* = 6, female) for the interventions planned by reference to the result of previous BFR study [21]. Consequently, it was determined that a minimum of 5-8 BFR training and 5-8 control participants were required to test both the main and interaction effects. Pre/Post effect sizes (ESs, Cohen's *d*) for muscle CSA, MVIC and 1RM were calculated using the following formula: [post mean - pre mean]/pre SD; *d* < 0.2 is a trivial effect, *d* = 0.2-0.5 is a small effect, *d* = 0.5-0.8 is a moderate effect, and *d* > 0.8 is a large effect [45].
